# SlTrxh functions downstream of SlMYB86 and positively regulates nitrate stress tolerance via S-nitrosation in tomato seedling

**DOI:** 10.1093/hr/uhae184

**Published:** 2024-07-10

**Authors:** Senlin Zeng, Xudong Sun, Jiali Zhai, Xixian Li, García-Caparrós Pedro, Hongjuan Nian, Kunzhi Li, Huini Xu

**Affiliations:** Faculty of Life Science and Technology, Kunming University of Science and Technology, Jingming South Street, Kunming, Yunnan 650224, China; Yunnan Key Laboratory of Crop Wild Relatives, The Germplasm Bank of Wild Species, Kunming Institute of Botany, Chinese Academy of Sciences, Kunming 650201, China; Faculty of Life Science and Technology, Kunming University of Science and Technology, Jingming South Street, Kunming, Yunnan 650224, China; Faculty of Life Science and Technology, Kunming University of Science and Technology, Jingming South Street, Kunming, Yunnan 650224, China; Department of Agronomy, University of Almeria, 04120, Almeria, Spain; Faculty of Life Science and Technology, Kunming University of Science and Technology, Jingming South Street, Kunming, Yunnan 650224, China; Faculty of Life Science and Technology, Kunming University of Science and Technology, Jingming South Street, Kunming, Yunnan 650224, China; Faculty of Life Science and Technology, Kunming University of Science and Technology, Jingming South Street, Kunming, Yunnan 650224, China

## Abstract

Nitric oxide (NO) is a redox-dependent signaling molecule that plays a crucial role in regulating a wide range of biological processes in plants. It functions by post-translationally modifying proteins, primarily through S-nitrosation. Thioredoxin (Trx), a small and ubiquitous protein with multifunctional properties, plays a pivotal role in the antioxidant defense system. However, the regulatory mechanism governing the response of tomato Trxh (SlTrxh) to excessive nitrate stress remains unknown. In this study, overexpression or silencing of *SlTrxh* in tomato led to increased or decreased nitrate stress tolerance, respectively. The overexpression of *SlTrxh* resulted in a reduction in levels of reactive oxygen species (ROS) and an increase in S-nitrosothiol (SNO) contents; conversely, silencing *SlTrxh* exhibited the opposite trend. The level of S-nitrosated SlTrxh was increased and decreased in *SlTrxh* overexpression and RNAi plants after nitrate treatment, respectively. SlTrxh was found to be susceptible to S-nitrosation both *in vivo* and *in vitro*, with Cysteine 54 potentially being the key site for S-nitrosation. Protein interaction assays revealed that SlTrxh physically interacts with SlGrx9, and this interaction is strengthened by S-nitrosation. Moreover, a combination of yeast one-hybrid (Y1H), electrophoretic mobility shift assay (EMSA), chromatin immunoprecipitation-quantitative PCR (ChIP-qPCR), and transient expression assays confirmed the direct binding of SlMYB86 to the *SlTrxh* promoter, thereby enhancing its expression. SlMYB86 is located in the nucleus and *SlMYB86* overexpressed and knockout tomato lines showed enhanced and decreased nitrate stress tolerance, respectively. Our findings indicate that SlTrxh functions downstream of SlMYB86 and highlight the potential significance of S-nitrosation of SlTrxh in modulating its function under nitrate stress.

## Introduction

Nitrogen (N) is an essential macronutrient for plant growth. The rates of nitrogen fertilizer supplies have experienced a significant surge, particularly in vegetable production under protected agricultural systems. Nevertheless, plants uptake less than 50% of the N fertilizer provided, while the remaining amount is either lost through leaching in the soil or discharged as agricultural runoffs [1]. Excess nitrate (NO_3_^−^) may be accumulated in soils and their quality deteriorates faster in greenhouse vegetable production than in conventional crop rotations [2]. Previous studies have reported substantial differences in ion accumulation between protected agricultural land and coastal areas. The soluble salt components, ranked in order of percentage, were as follows: NO_3_^−^ > SO_4_^2−^ > Ca^2+^ > Cl^−^ > Na^+^, based on mass [3]. Greenhouse soils often accumulate nitrates in higher levels, which can pose environmental hazards and result in a decline in the quality of vegetables [4]. Regarding plant physiological issues, NO_3_^−^ excess caused oxidative stress in spinach [5], limited cucumber plant development [6], modifications in the composition of the cell as well as in its structure, and patterns of genes expression linked to lignin synthesis in *Brassica napus* [7]. Furthermore, nitrate excess altered the accumulation of proteins linked to photosynthetic processes, stress and oxidative damage responses [8].

Nitric oxide (NO) is a gaseous molecule and a crucial biological messenger that plays a pivotal role in various plant physiological processes, including morphogenesis, development, and tolerance to biotic and abiotic stress conditions [9–12]. NO primarily functions through S-nitrosation [13, 14]. This process involves the covalent attachment of a NO molecule to a cysteine thiol group on a protein or peptide, leading to the formation of S-nitrosothiols (SNOs). The level of S-nitrosation of cellular protein is linked to the equilibrium between S-nitrosation and denitrosation. The main protein denitrosylases in human cells are glutathione (GSH), S-nitrosoglutathione reductase (GSNOR), and thioredoxin (Trx). The role of GSH as a denitrosylating agent was well established [15]. The natural NO donor, S-nitrosoglutathione (GSNO), synthesized by the reaction of NO with GSH, acts as a reservoir of NO bioactivity [16]. GSNOR influences the bioavailability of NO via GSNO dissociation, regulates NO signaling and modulates the overall levels of S-nitrosylated proteins in cells [17–20]. In mammalian cells, Trx participates in the maintenance of the redox homeostasis and several physiological processes via disulfide reduction, S-nitrosation/S-denitrosation reactions and protein–protein interactions [21]. The S-nitrosation of Trx1 is essential for scavenging ROS and maintaining redox regulatory activity [22]. S-nitrosation of Trx also plays a role in the anti-apoptotic function of Trx [23].

Trx is an antioxidant enzyme widely distributed, highly conserved with a low molecular weight (14 kDa) and a common redox active site (WCGPC). Trx is a disulfide oxidoreductase able to control the activity of enzymes via breakdown of their disulfide bridge. Thioredoxin (Trx) plays crucial roles in maintaining redox homeostasis at the cellular level by sensing and transferring reducing equivalents to various target proteins [24]. Subsequently, oxidized Trxs are reduced by thioredoxin reductase. In *Arabidopsis thaliana*, Trxs are classified into six distinct groups: Trx h, Trx f, Trx m, Trx x, Trx y, and Trx o, distributed within various subcellular compartments including chloroplasts, mitochondria, and the cytoso [25]. The genome of *A. thaliana* encodes nine h-type Trx proteins, predominantly situated in the cytosol. Trx-mediated oxidative processes play a pivotal role in signal transmission during biotic and abiotic stress conditions. Specifically, h-Type Trx is involved in tobacco defense responses against two virus species and various abiotic stressors [26]. *MaTrx12* improved the chilling tolerance of harvested banana fruit by regulating redox homeostasis [27]. The expression of *A. thaliana* thioredoxin-h2 in *B. napus* decreased oxidative damage and improved salt tolerance [28].

Tomato (*Solanum lycopersicum*) is a highly important crop grown on a global scale [29]. According to projections, global tomato production is expected to increase from 41.52 million tons in 2020 to 51.93 million tons in 2026, assuming the standard tomato production scenario [30]. Due to its importance as horticultural crop, it has been bred to improve productivity, fruit quality, and resistance to stress [31]. Our previous research showed that overexpressed lines of *SlTrxh* in tobacco enhanced the tolerance to nitrate-induced stress [32]. To gain a deeper understanding of the mechanism of the tomato *SlTrxh* under nitrate stress, we conducted research to investigate the responses of *SlTrxh* overexpression and RNAi tomato plants to excess nitrate stress. Additionally, experiments utilizing yeast two-hybrid (Y2H), co-immunoprecipitation (Co-IP), and luciferase complementation assay (LCA) techniques confirmed the interaction between SlTrxh and SlGrx9. To characterize the regulation of *SlTrxh* by SlMYB86, yeast one-hybrid (Y1H), electrophoretic mobility shift assay (EMSA), transient expression assay, and chromatin immunoprecipitation-quantitative PCR (ChIP-qPCR) were employed. The impact of SlMYB86 overexpression and knockout in tomatoes under nitrate stress was also investigated. Consequently, our findings contribute novel insights into the roles of tomato *SlTrxh* and *SlMYB86* in the response to nitrate stress.

## Results

### SlTrxh positively regulates the excess nitrate stress tolerance in tomato seedlings

**Figure 1:**
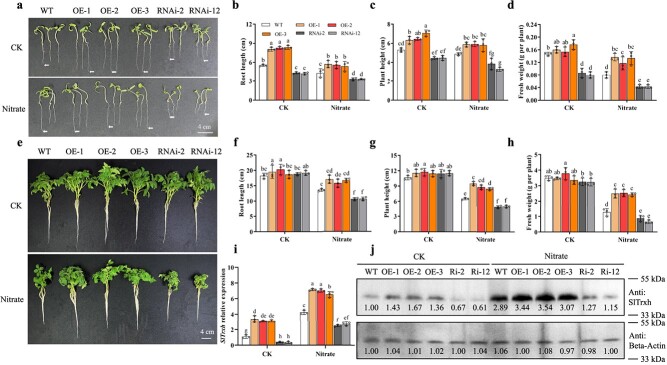
*SlTrxh* positively regulates the excess nitrate stress tolerance in tomato. **a** Photographs of *SlTrxh* overexpression (OE) and RNAi lines grown on wet filter paper with or without excess nitrate. The scale bar represents 4 cm. **b** Root length of plants in **a**. **c** Plant height of tomatoes in **a**. **d** Fresh weight of tomatoes in **a**. **e** Photographs of 2-week-old *SlTrxh* OE and RNAi plants after 20 days of excess nitrate treatment. **f** Root length of seedlings in **e**. **g** Plant height of seedlings in **e**. **h** Fresh weight of seedlings in **e**. **i** Quantitative RT-PCR analysis of *SlTrxh* expression in WT and *SlTrxh* transgenic plants. **j** SlTrxh protein level in WT, OE, and RNAi plants. The quantification of the data, carried out using the ImageJ software, is shown below the blot. The lowercase letters a, b, c, and d are used to indicate significant variations among different groups or conditions at a significance level of *P* < 0.05. Data represent the mean ± SE (*n* = 3).

To investigate the role of *SlTrxh* in tomatoes under nitrate-induced stress conditions, we developed lines with overexpressed *SlTrxh* (OE) and RNA interference (RNAi) lines (Fig. S1, see online supplementary material). After 7 days of excess nitrate treatment, the OE lines exhibited longer root lengths compared to wild type (WT) plants, whereas the RNAi lines displayed shorter root lengths (Fig. 1a and b). Furthermore, the OE and RNAi lines displayed a similar trend to root length in term of height and fresh weight when compared to WT plants (Fig. 1c and d). The data suggest that *SlTrxh* positively regulates the tolerance of tomato seedlings to excess nitrate stress.

In order to further investigate the role of *SlTrxh* in tomato seedlings, 2-week-old transgenic tomato plants overexpressing and RNAi were subjected to excess nitrate treatment. After excess nitrate treatment, the overexpression lines exhibited enhanced growth compared to the WT, manifested through longer root length, increased plant height, and higher fresh weight. In contrast, the growth of RNAi tobacco was notably suppressed in comparison to the wild type (Fig. 1e–h). In the controlled conditions, the expression levels of *SlTrxh* were significantly higher in overexpression lines compared to the WT, whereas its expression was lower in RNAi plants. Following nitrate treatment, the expression of *SlTrxh* increased in all lines. Notably, in comparison to the WT, the expression of *SlTrxh* was elevated in overexpression lines and reduced in RNAi lines (Fig. 1i). The SlTrxh protein level in RNAi plants was lower than WT in both control and nitrate stressed conditions (Fig. 1j). These data reported that the tolerance of tomato to excess nitrate stress may be related to the expression level of *SlTrxh*.

**Figure 2:**
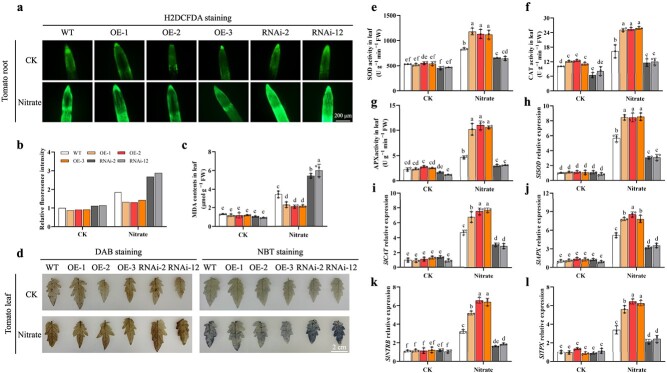
Reactive oxygen species scavenging capacity of *SlTrxh* overexpression and RNAi tomato plants under excess nitrate stress. The tomato seedlings, aged two weeks, were subjected to irrigation with either a 10 mM solution of nitrate (control) or a 100 mM nitrate solution for a period of 20 days. **a** ROS dye H_2_DCFDA staining of primary root tips. Scale bar is 200 μm. **b** The relative fluorescence intensity of **a** was carried out using the ImageJ software. **c** MDA contents. **d** H_2_O_2_ and O_2_^**·**−^ histochemical staining with DAB (left) and NBT (right). **e**–**l** activities of antioxidant enzymes of SOD (e), CAT (f), APX (g) and expression of antioxidant enzymes of *SlSOD* (h), *SlCAT* (i), *SlAPX* (j), *SlNTRB* (k), *SlTPX* (l) in tomato leaves. Data represent the mean ± SE (*n* = 3).

Given the role of SlTrxh as an active antioxidant, we assessed the levels of reactive oxygen species (ROS) in WT plants, those with *SlTrxh* overexpression, and plants in which *SlTrxh* expression was inhibited (RNAi) with and without nitrate treatment. This was done using the fluorescent dye 2′,7′-dichlorofluorescin diacetate (H2DCFDA). Under nitrate stress, the ROS contents were higher in RNAi plants and lower in OE plants, compared with WT (Fig. 2a and b). Malondialdehyde (MDA) serves as a prevalent indicator of oxidative lipid damage resulting from exposure to environmental stressors. The nitrate stress treatment significantly enhanced the MDA content in *SlTrxh* RNAi plants, while the content in OE was lower than WT (Fig. 2c). The result of histochemical staining with DAB and NBT showed that H_2_O_2_ and O_2_^**·**−^ contents have the same trend with MDA contents (Fig. 2d; Fig. S2, see online supplementary material). The activities of the antioxidant enzymes SOD, CAT, and APX, which function to scavenge excess ROS, were assessed in this study. The enzyme activities did not show any notable variations in the control treatment (CK). After the application of nitrate stress treatment, enzyme activities in all tomato plants exhibited an increase. Nevertheless, the enzyme activities in the RNAi lines were considerably lower compared to those in the WT plants when exposed to excessive nitrate stress (Fig. 2e–g). In addition, the transcript levels of *SlSOD*, *SlCAT*, *SlAPX*, *SlNTRB*, and *SlTPX* in OE were higher than WT and lower in RNAi tomato plants after nitrate treatment (Fig. 2h–l). These findings indicated that SlTrxh induced ROS scavenging through a set of antioxidant enzymes.

### S-nitrosation is important for the function of SlTrxh under nitrate stress

**Figure 3:**
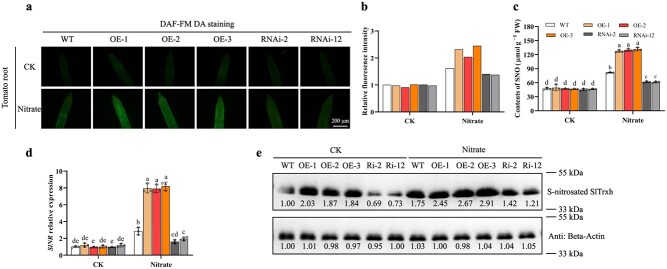
S-nitrosation is important for the function of SlTrxh under nitrate stress. The tomato seedlings, aged two weeks, were subjected to irrigation with either a 10 mM solution of nitrate (control) or a 100 mM nitrate solution for a period of 20 days. **a** The impact of excessive nitrate stress on the accumulation of NO—NO dye DAF-FM staining of primary root tips. Scale bar is 200 μm. NO accumulation was done with 10 μM DAF-FM in 10 mM Tris–HCl in the root tips of the main root. **b** The relative fluorescence intensity of **a** was carried out using the ImageJ software. **c** SNOs contents. **d**  *SlNR* mRNA transcript level. **e** S-nitrosated SlTrxh level in *SlTrxh* overexpression and RNAi transgenic tomato seedlings. The quantification of the data, which was carried out using the ImageJ software, is shown below the blot. Data represent the mean ± SE (*n* = 3).

NO is a crucial mediator that works in cooperation with ROS. Then, the NO accumulation in *SlTrxh* transgenic tomato seedlings after nitrate stress treatment was analysed. As shown in Fig. 3a and b, the NO accumulation did not show obvious variation between WT, OE, and RNAi lines in the CK. After nitrate treatment, there was an increase in NO accumulation in overexpressing lines (OE) and a decrease in NO content in SlTrxh knockdown lines, as compared to the WT. Likewise, following nitrate stress treatment, the levels of SNOs significantly rose in OE1, OE2, and OE3, but declined in the RNAi lines compared to the WT (Fig. 3c). The expression of *SlNR* in OE and RNAi lines showed the same trend with SNOs content (Fig. 3d). These results suggests that NO accumulation is important for the nitrate stress tolerance of *SlTrxh* transgenic tomato seedlings.

Besides, the S-nitrosated level of SlTrxh was investigated in WT, OE, and RNAi lines. As shown in Fig. 3e, the S-nitrosated level of SlTrxh in RNAi line was reduced compared to WT under normal conditions. After nitrate treatment, although the S-nitrosated level of SlTrxh in all genotypes was increased, the level in RNAi tomato was still lower than WT, suggesting that silencing of *SlTrxh* leads to decreased NO contents and S-nitrosation level of SlTrxh under nitrate stress. These results therefore highlight the crucial role of S-nitrosated level of SlTrxh in nitrate stress tolerance.

**Figure 4:**
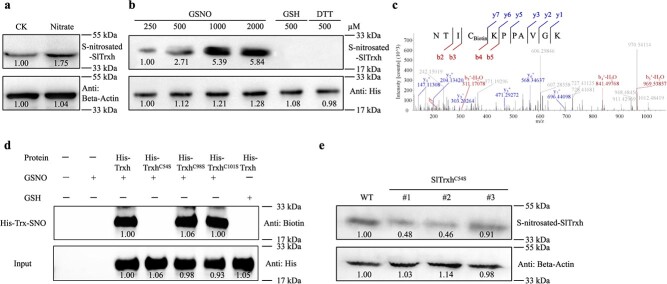
S-nitrosation analysis of SlTrxh *in vivo* and *in vitro*. **a** S-nitrosated levels of SlTrxh in tomato leaves treated with excess nitrate. Tomato seedlings were treated with 10 mM of nitrate (CK) or 100 mM of nitrate for 24 hours. 100 ng total protein of tomato leaves were loaded per lane and subjected for Biotin Switch Test (BST). **b** 10 ng purified recombinant Trxh protein was treated with increased concentrations of GSNO (250, 500, 1000, 2000 μM GSNO, 500 μM GSH, or 500 μM DTT) and underwent BST. GSH and DTT were served as negative controls. Quantification of the data is shown below the blot. **c** Mass spectrometric analyses identified Cys54 as the S-nitrosated site in the SlTrxh protein. The MS/MS spectra of Cys54 originated from a biotin-charged SlTrxh peptide (NTICKPPAVGK). **d** Effects of Cys 54, 98, and 101 to Ser site-directed mutation on S-nitrosation of SlTrxh upon GSNO treatment. 10 ng of recombinant SlTrxh protein were loaded per lane. Quantification of the data is shown below the blot. **e** S-nitrosation of SlTrxh in SlTrxh^C54S^ overexpressed transgenic tobacco. #1, #2, and #3 refer to different tobacco transgenic lines in which the amino acid Cys54 of SlTrxh has been substituted with Ser. 100 ng total protein of tobacco leaves were loaded per lane. Quantification of the data is shown below the blot.

The S-nitrosated SlTrxh protein in tomato increased under nitrate stress (Fig. 4a), highlighting that SlTrxh was S-nitrosated *in vivo* under excess nitrate stress. Then, the purified pET28a-SlTrxh protein treatment with 250, 500, 1000, and 2000 μM GSNO was investigated whether SlTrxh could be S-nitrosated *in vitro*. The result showed that the S-nitrosated SlTrxh was increased with the increasing GSNO concentration. Besides, S-nitrosated SlTrxh was not detected with GSH or DTT treatment (Fig. 4b). These results indicated that SlTrxh could be S-nitrosated both *in vivo* and *in vitro*.

S-nitrosation usually occurs on Cysteine (Cys) residues of proteins. To obtain the S-nitrosated site, mass spectrometric analyses were performed on the His-SlTrxh recombinant protein. The Cys54 of SlTrxh, labeled with biotin, was identified as the S-nitrosated site (Fig. 4c). To validate this conclusion, we analysed the S-nitrosation sites of SlTrxh *in vitro* using point mutations. The SlTrxh protein contains three Cys situated at positions 54, 98, and 101. The Cys 54 was mutated to Serine (SlTrxh^C54S^), which is structurally similar to cysteine and is used as a non-nitrosylatable mutation. The other two sites were treated in the same way. As shown in Fig. 4d, the S-nitrosation signal completely disappeared in the SlTrxh^C54S^; however, it did not appear to be affected in SlTrxh^C98S^ and SlTrxh^C101S^.

We then analysed whether Cys 54 is important for the S-nitrosated level of SlTrxh using the *SlTrxh^C54S^* overexpressing tobacco plants [32]. Upon mutation of this site, the tobacco’s capacity to withstand nitrate stress is significantly diminished (Fig. S3, see online supplementary material). This reduction is conspicuously manifested in the mutant’s shorter root length, accumulation of more ROS, and lower NO contents than WT and overexpression plants. The S-nitrosated level of SlTrxh^C54S^ markedly decreased in the overexpressing SlTrxh^C54S^ mutant plants compared to the WT tobacco plant (Fig. 4e). These results suggest that Cys54 is a critical site for SlTrxh’s response to nitrate stress.

### Interaction of SlTrxh and SlGrx9 was enhanced by S-nitrosation

**Figure 5:**
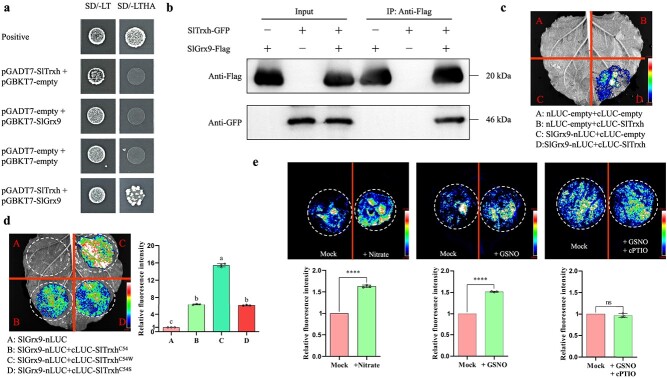
**SlTrxh physically interacts with SlGrx9**. **a** Y2H assay showing interactions of SlTrxh and SlGrx9. Yeast cells with pGBKT7-SlGrx9 and pGADT7-SlTrxh were grown well both on SD/−Leu/−Trp and SD/−Leu/−Trp/-His/−Ade mediums. Positive control containing pGADT7-T + pGBKT7-53 and pGADT7-SlTrxh + BD, AD + pGBKT7-SlGrx9 and AD + BD were served as negative controls. **b** Co-IP assay showing SlTrxh and SlGrx9 interaction. *SlTrxh* and *SlGrx9* were tagged with the GFP and Flag tags. The precipitate was detected using anti-GFP and anti-Flag antibodies. **c** Biomolecular luciferase complimentary assay (LCA) showing the interaction of SlTrxh and SlGrx9. **d** and **e** S-nitrosation enhances the interaction between SlTrxh and SlGrx9. The 54th cysteine residue of SlTrxh mutated into tryptophan (which is proposed to undergo nitrosation) or serine (which is proposed to lose nitrosation). Nitrate and GSNO were added in the LCA experiment. GSNO acted as a NO donor and cPTIO was used as a NO scavenger. The relative quantification was carried out using the ImageJ software.

To identify the interacting partner of SlTrxh, the yeast two-hybrid (Y2H) assay was utilized to screen a tomato cDNA library. The tomato glutaredoxin (SlGrx9) (GenBank Accession No. NM_001323560.1) was identified as a probably interacting protein of SlTrxh. Then, we performed independent Y2H assay to confirm the interaction of SlTrxh with SlGrx9. The results showed that SlTrxh interacted with SlGrx9 in yeast cells (Fig. 5a). The Co-immunoprecipitation (CoIP) assays, conducted by co-expressing 35S: SlTrxh-GFP and 35S: SlGrx9-Flag in *Nicotiana benthamiana* leaves, revealed the *in vivo* immunoprecipitation of SlTrxh-GFP by SlGrx9-Flag (Fig. 5b). Moreover, to ensure the interaction of SlTrxh and SlGrx9 in planta, we then carried out the bimolecular LCA. *N. benthamiana* leaves co-expressing cLUC-SlTrxh and SlGrx9-nLUC had strong luciferase activity. Nevertheless, the negative control group did not show any luciferase activity (Fig. 5c). These findings suggested that SlTrxh physically interacted with SlGrx9.

**Figure 6:**
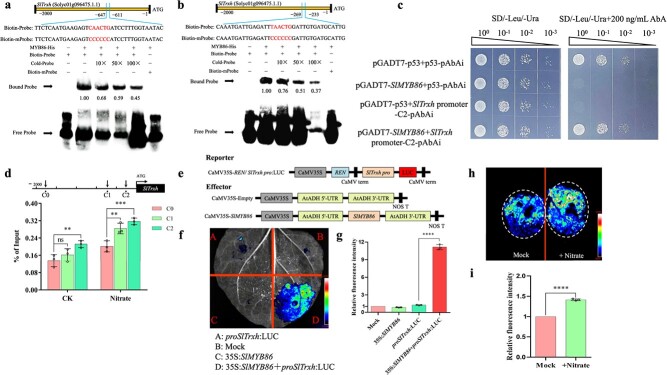
SlMYB86 directly activates the expression of *SlTrxh*. **a**–**b** Electrophoretic mobility shift assay (EMSA) performed to investigate the binding of SlMYB86 to the promoter region of SlTrxh, which contains the CAACTG/TAACTG motif. **c** Yeast one-hybrid (Y1H) system of SlMYB86 binding to *SlTrxh* promoter. **d** Chromatin immunoprecipitation-quantitative PCR (ChIP-qPCR) assay of the enrichment of SlMYB86 activating *SlTrxh* promoter. Here, C0 was served as a negative control. **e** A schematic diagram of the dual-luciferase reporter sassy with reporter and effector vector. **f** Dual-luciferase reporter assay. **g** Relative quantification of **f** was carried out using the ImageJ software. **h** Dual-luciferase reporter of the transcription level of SlMYB86 on *SlTrxh* with nitrate. **i** Relative quantification of **f**.

Meanwhile, we found that mutating the 54th cysteine residue of SlTrxh to Tryptophan (which is proposed to undergo nitrosation) could enhance the interaction between the SlTrxh and SlGrx9 (Fig. 5d). To investigate if excess nitrate and S-nitrosation affect the interaction between SlTrxh and SlGrx9, nitrate and GSNO was added in the LCA. Nitrate and GSNO enhanced the interaction between SlTrxh and SlGrx9, while cPTIO decreased the interaction (Fig. 5e), suggesting that S-nitrosation enhanced the interaction of SlTrxh and SlGrx9.

### SlMYB86 acts upstream of SlTrxh

Our previous RNA-sequencing data revealed a co-expression pattern between the transcription factor *SlMYB86* and *SlTrxh* [32]. There is a possibility that SlMYB86 regulates the expression of *SlTrxh*. To check the hypothesis, we tested the 2-kb promoter fragment of *SlTrxh* using PlantCARE (http://bioinfor-matics.psb.ugent.be/webtools/plantcare/html/) (Fig. S4, see online supplementary material). Two MYB binding sites (CAACTG and TAACTG), named C1 and C2, were identified in the promoter of *SlTrxh*. Later, we carried out an EMSA using recombinant SlMYB86 in *Escherichia coli*. SlMYB86 bound to the MYB binding site of the *SlTrxh* promoter (Fig. 6a and b). Then, the yeast one-hybrid (Y1H) system was used to determine whether SlMYB86 could bind to *SlTrxh* promoter. As shown in Fig. 6c, all of the yeast transformants grew well on SD/−Leu/-Ura medium while only the positive control (pGADT7-p53 + p53-pAbAi) and those transformants containing pGADT7-SlMYB86 *+ SlTrxh* promoter-C2-pAbAi could grow in a normal manner normally on SD/−Leu/-Ura/+200 ng/mL AbA medium. These findings noted that SlMYB86 could ligate to the promoter of *SlTrxh in vitro.* ChIP-qPCR assay was performed to assess whether SlMYB86 binds to C1 and C2 motifs *in vivo*. The results demonstrated that the C2 region of the promoter was more significantly enriched than C1 in the *35S:SlMYB86-GFP* samples after nitrate treatment (Fig. 6d). A transient expression assay was performed to confirm whether SlMYB86 could activate the expression of *SlTrxh*. The results showed that co-injection of *35S:SlMYB86* and *ProSlTrxh:*LUC in tobacco leaves enhanced fluorescence signal compared to injection of *ProSlTrxh:LUC* in tobacco leaves (Fig. 6e–g). After injecting excess nitrate into tobacco leaves for 6 hours, the fluorescence signal was significantly enhanced (Fig. 6h and i). These findings showed that SlMYB86 can directly ligate to the *SlTrxh* promoter to activate its expression.

### SlMYB86 positively regulates excess nitrate tolerance of tomato seedlings

**Figure 7:**
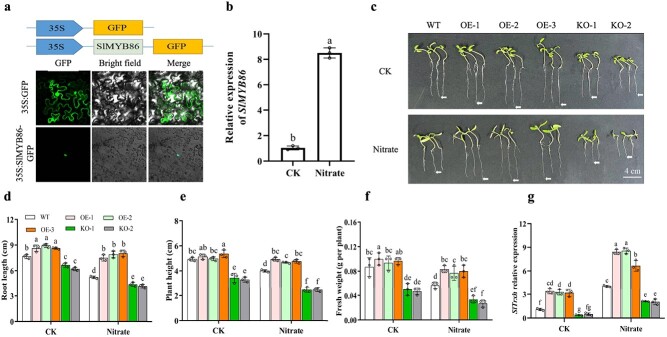
*SlMYB86* exhibits a positive role under nitrate stress conditions. **a** Subcellular localization of SlMYB86 in tobacco leaf epidermal cells. **b** Expression of *SlMYB86* in tomato root under nitrate stress by qRT-PCR. **c** Phenotype of *SlMYB86* overexpression transgenic tomato seedlings under nitrate stress. Scale bar is 4 cm. **d**–**f** Root length, plant height ,and fresh weight of OE and KO tomato seedlings under nitrate stress. **g** The expression level of *SlTrxh* in OE and KO plants of *SlMYB86* under nitrate stress. Data represent the mean ± SE (*n* = 3).

To characterize the function of *SlMYB86* in tomato under nitrate stress, we reconstructed the *35S:SlMYB86-GFP* vector. Laser confocal microscopy showed the SlMYB86 protein was localized in the nucleus (Fig. 7a), suggesting it might function as a transcription factor. The relative expression of *SlMYB86* in the tomato root was 8.5-fold of the CK after nitrate stress (Fig. 7b). Then, *SlMYB86* overexpression tomato (OE1-3) and CRISPR-Cas9 knockout (KO-1, KO-2) plants were generated (Figs S5 and S6, see online supplementary material). As shown in Fig. 7c, after 7 days of treatment with excess nitrate, the three overexpression lines showed significant higher tolerance compared to the two knockout lines in terms of root length, plant height, and fresh weight (Fig. 7d–f). Furthermore, the expression level of *SlTrxh* was examined in both overexpressed and knock-out plants of *SlMYB86*. As shown in Fig. 7g, the expression of *SlTrxh* was elevated in *SlMYB86* OE lines compared to WT, while it was reduced in KO plants under both CK and nitrate stress conditions. These findings demonstrate that *SlMYB86* positively regulates tomato tolerance to excess nitrate stress, with the involvement of *SlTrxh*.

## Discussion

Secondary salinization, resulting from the excessive accumulation of calcium and potassium nitrate in soils due to over-fertilization, has emerged as a significant challenge in protected vegetable production. Excess nitrate increased the levels of ROS and membrane peroxidation in tomato plants [33]. ROS over-accumulation can result in oxidative stress and the degradation of macromolecules such as polysaccharides, proteins, nucleic acids, and lipids. In plants, the production of ROS is tightly regulated by an efficient antioxidative system that comprises both enzymatic and non-enzymatic compounds. The enzymes responsible for scavenging ROS comprise CAT, SOD, and POD. Furthermore, the enzymes participating in the ascorbate-glutathione (AsA-GSH) cycle include monodehydroascorbate reductase (MDHAR), dehydroascorbate reductase (DHAR), ascorbate peroxidase (APX), and glutathione reductase (GR) [34]. The cell possesses various methods to avoid oxidative damage, such as Trx and glutathione systems [35]. Trxs act as antioxidants by modulating the redox states of target proteins through cysteine thiol-disulfide exchanges, thereby playing a pivotal role in regulating the scavenging of ROS. The study revealed that *SlTrxh* positively impacts tomato’s tolerance to excess nitrate stress, and we delved into the associated molecular mechanisms.

### S-nitrosation modification is important for the function of SlTrxh under nitrate stress

The Trx system, consisting of Trx protein, Trx reductase, and NADPH, plays a vital role as a disulphide reductase system found in all living organisms. The Trx system in mammalian cells employs thiol and selenol groups to uphold a reducing intracellular environment for counteracting oxidative/nitrosative stress. The function of Trx is well documented under abiotic stress. *Lobularia maritima* transgenic tobacco lines overexpressing *LmTrxh2* exhibited heightened tolerance to salt and osmotic stress comparison to non-transgenic plants. This enhanced tolerance was attributed to the reduction of oxidative damage [36]. Trx CDSP32 alleviated the Cd-induced photo inhibition in tobacco leaves [37]. In our study, the growth of *SlTrxh* overexpression and RNAi transgenic tomato was better and worse than WT, respectively. Overexpressing *Tamarix hispida* Trx5 (ThTrx5) in *Arabidopsis* triggers stress response pathways, leading to enhanced salt tolerance. Under salt stress conditions, transgenic plants with *ThTrx5* showed notably increased activities of SOD, POD, and CAT, as well as higher fresh weight compared to WT plants [38]. The upregulation of Cu/Zn-SOD and Mn-SOD was observed in tobacco plants when *Medicago sativa* Trx was overexpressed under salt stress conditions [39]. In this experiment, we observed that the expression levels of *SlSOD, SlCAT, SlAPX, SlNTRB*, and *SlTPX* in RNAi tomato plants were significantly reduced compared to WT plants following nitrate treatment. Consequently, RNAi-modified tomato plants exhibit elevated levels of ROS under nitrate stress, likely due to decreased activity and expression of antioxidant enzymes. This observation underscores the pivotal role of *SlTrxh* in modulating tomato plant tolerance to nitrate stress by mitigating oxidative damage.

S-nitrosation of chloroplastic thioredoxin M2 enhances plant defense mechanisms in response to water deficiency [40]. An important feature of Trx1 is its regulation through oxidation and S-nitrosation of its various Cysteine residues within redox environments [41]. This study demonstrates that Cys54 of SlTrxh plays a pivotal role as a site for S-nitrosation under high levels of nitrate.

NR is essential for NO synthesis [42]. The accumulation of NO and the expression of SlNR significantly decreased in RNAi tomato plants following nitrate stress treatment compared to the WT. The SNOs accumulation decreased more in RNAi tomato plants after nitrate stress indicating that the S-nitrosation level was lower in RNAi tomato plants. The S-nitrosated level of SlTrxh was increased after nitrate stress and the S-nitrosated SlTrxh level in RNAi plants was lower than WT, suggesting that S-nitrosation of SlTrxh might regulate NO production in response to nitrate stress tolerance.

### SlGrx9 physically interacts with SlTrxh

Glutaredoxins (Grxs) are versatile proteins involved in redox reactions with small size. They display oxidoreductase activity that depends on glutathione, glutathione reductase, and NADPH [43]. Our research employed Y2H, Co-IP, and LCA experiments to validate the interaction of SlTrxh and SlGrx9. Furthermore, we found that treatment with nitrate and GSNO significantly enhanced this interaction. Overexpression of *Arabidopsis* monothiol glutaredoxin *AtGRXS17* in chrysanthemum enhanced heat stress response [44]. Similarly, *OsGRX20* has a positive role in enhancing rice’s tolerance to various stressors [45]. In *A. thaliana*, knockout of *Grx9S15* slowed plant respiration, reduced plant tolerance to arsenic, and affected plant growth and development [46]. The function of *SlGrx9* under nitrate stress needs further study.

### The positive regulation of SlMYB86 on *SlTrxh* enriches the regulatory network of excess nitrate stress tolerance

The MYB family is one of the most extensive transcription factor families in plants, with MYB proteins playing a crucial role in regulating plant responses to diverse stresses [47, 48]. The R2R3-type MYB gene *SlMYB102* in transgenic tomatoes enhances salt tolerance by decreasing ROS production compared to the WT [49]. Overexpression of ThMYB8 in Arabidopsis plants resulted in a notable reduction in the levels of O_2_^**·**−^, H_2_O_2_, and MDA compared to the WT plants under saline conditions [50]. Overexpression of *PsnMYB108* may enhance tobacco’s salt stress tolerance by increasing its ROS scavenging capability [51]. The overexpression of *AtMYB49* in *Arabidopsis* led to an increased antioxidant capacity through the up-regulation of genes encoding peroxidases and late embryogenesis abundant proteins (LEAPs) [52]. *TaMYB86B*, through the regulation of ion homeostasis, plays a crucial role in enhancing wheat’s salt tolerance by maintaining an appropriate osmotic balance and reducing levels of ROS [53]. Subcellular localization within the nucleus indicates that SlMYB86 likely functions as a transcription factor (TF), which regulates downstream gene expressions by binding to specific *cis*-acting elements in the promoter region. Our research presents robust evidence from both *in vitro* and *in vivo* studies supporting the direct binding of SlMYB86 to the promoter region of *SlTrxh*, thereby enhancing its expression. Tomato lines with increased expression of *SlMYB86* show enhanced tolerance to nitrate stress compared to the WT, while *SlMYB86* knockout lines exhibit reduced tolerance to nitrate stress. These findings underscore the crucial role of SlMYB86 in modulating nitrate stress tolerance through the regulation of *SlTrxh* expression.

**Figure 8:**
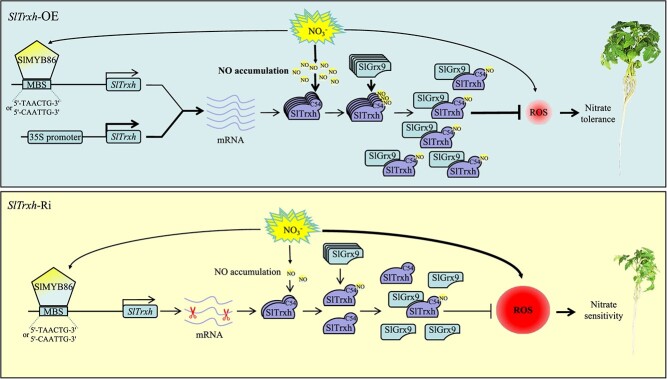
A schematic illustration of *SlTrxh* positively regulates nitrate stress tolerance through S-nitrosation in tomato. In the *SlTrxh* overexpression plants, SlMYB86 directly binds to the promoter of SlTrxh, thereby activating its expression. The NO accumulation and the S-nitrosation of SlTrxh were induced under nitrate stress, with Cys54 identified as a crucial site for this process. The S-nitrosation enhances the interaction between SlTrxh and SlGrx9, thereby improving tomato’s resistance to nitrate stress with lower ROS accumulation. While in the *SlTrxh* RNAi plants, the expression and S-nitrosated level of SlTrxh was reduced and the interaction of SlTrxh and SlGrx9 was lower than overexpression plants under nitrate stress with more ROS accumulation.

Through comprehensive phenotypic and molecular analyses, we have proposed a possible working model for SlTrxh in response to excessive nitrate stress (Fig. 8). In the *SlTrxh* overexpression plants, SlMYB86 directly binds to the promoter of *SlTrxh*, thereby activating its expression. Nitrate stress induced the NO accumulation and the S-nitrosation of SlTrxh, with Cys54 identified as a crucial site for this process. The S-nitrosation enhances the interaction between SlTrxh and SlGrx9, thereby improving tomato’s resistance to nitrate stress. While in the *SlTrxh* RNAi plants, the expression and S-nitrosated level of SlTrxh was reduced and the interaction of SlTrxh and SlGrx9 was lower than overexpression plants under nitrate stress. Collectively, this study elucidates a novel molecular mechanism by which *SlTrxh* is regulated by SlMYB86 and S-nitrosation of SlTrxh positively regulates nitrate stress tolerance.

## Materials and methods

### Plasmid construction and transgenic tomato transformation

The genetic sequence of SlTrxh (GenBank Accession No. NM_001247540.2) was amplified and then inserted into the RNAi vector pHELLSGATE8 (Invitrogen, California, USA). PCR amplification was used to acquire the genetic sequences of *SlTrxh* and *SlMYB86* (GenBank Accession No. XM_004232564.4), which were subsequently subcloned into the plasmid pCAMBIA1300 with the ClonExpress II one-step cloning kit from Vazyme Biotech Co., Ltd. Knockout vectors targeting SlMYB86 were generated using the CRISPR-Cas9 technique as described by Kim *et al.* [54], with some modifications. In summary, the primers for the target gene were submitted on the CRISPR-Cas9 primer design website (http://crispr.hzau.edu.cn/cgi-bin/CRISPR2/CRISPR), using pHEE401E-pME6DT1DT2 as the template for PCR amplification. The resulting amplified band, approximately 600–700 bp in size, was then combined with the pHEE401E vector for subsequent digestion, ligation, and transformation processes. Following this, single colonies were chosen for bacterial liquid PCR, and samples were sent for testing and validation of the desired target. For a full list of primers, refer to Table S1 (see online supplementary material).

Recombinant plasmids were introduced into the *Agrobacterium tumefaciens* strain LBA4404 and subsequently employed to transform Ailsa Craig (AC) tomatoes. The confirmation of overexpression transgenic plants involved genomic PCR, qRT-PCR, or Western blot analysis. Genomic PCR was used for the characterization of transgenic CRISPR/Cas9 plants, which were subsequently subjected to DNA sequencing.

### RNA extraction and qRT-PCR analysis

RNA extraction and qRT-PCR analyses were carried out using the Trizol reagent (Takara, China). Specific primer information is available in Table S2 (see online supplementary material). The 2^−ΔΔCt^ method was applied for quantifying the relative expression levels of different genes, with a minimum of three biological replicates conducted.

### Protein extraction and Western blot analysis

The process of extracting proteins and Western blot assays was carried out in accordance with the previously established protocol [55]. Proteins were separated using 12% gradient SDS-PAGE gels, transferred onto PVDF membranes, and then blocked for 1 hour in a blocking solution containing 1% nonfat milk and 0.5% Tween 20 in TBS. The membranes were subsequently incubated overnight with primary antibodies, namely Anti-Biotin (Cell Signaling Technology, 7075 s), Anti-GFP (Clontech, 632 381), Anti-Flag (Beyotime, AF519), Anti-His (Beyotime, AH367), Anti-Actin (Proteintech, 60 008-1-Ig), and Anti-SlTrxh [32]. In this study, the secondary antibodies employed were Goat Anti-Mouse IgG1, Fc gamma Specific Antibody (Cell Signaling Technology, 96714S), and Anti-Mouse IgG, HRP-linked Antibody (Cell Signaling Technology, 7076S). Western blot (WB) band gray values were analysed using the ImageJ software (National Institutes of Health, Bethesda, MD, USA).

### Excess nitrate stress treatments

Tomato seeds were soaked in water at 55°C for 60 to 120 minutes. Following this treatment, the seeds were placed on a petri dish lined with two layers of moist filter paper and germinated at 28°C for a period of 2 days. Subsequently, the germinated seeds were transferred onto filter paper saturated with either water (control) or a 100 mM nitrate solution (where KNO_3_ and Ca(NO_3_)_2_ offered an equivalent concentration of NO_3_^−^) for 7 days before being photographed. Transgenic and WT tomato seedlings were watered with either 0 mM or 100 mM nitrate solution (KNO_3_ and Ca(NO_3_)_2_ provided an equal concentration of NO_3_^−^) for 20 days. The control group (CK) was watered with regular water. The plants were harvested and stored at −80°C for subsequent analysis.

### NBT and DAB histochemical staining

Superoxide radicals (O_2_^·−^) content was analysed histochemically staining with nitroblue tetrazolium (NBT) [56]. Levels of H_2_O_2_ were histochemically stained with diaminobenzidine (DAB) [57].

### MDA and H_2_O_2_ contents assays

The malonaldehyde (MDA) contents were assayed using the thiobarbituric acid reaction method [58]. H_2_O_2_ levels were estimated based on the protocol described by Gay and Gebicki [59].

### Determination of antioxidant enzyme activity

Leaf samples weighing 0.2 g were homogenized in 2 mL of chilled extraction buffer (pH 7.0) containing 1% polyvinylpyrrolidone (PVP) and 0.1 mM EDTA using a mortar and pestle in an ice bath. Following centrifugation (12 000 *g*, 20 min, 4°C), the resultant supernatant was utilized for assessing the activities of antioxidant enzymes. The spectrophotometric evaluation of superoxide dismutase (SOD) activity was conducted at 560 nm [60]. The activity of catalase (CAT) was determined by measuring the reduction in absorbance at 240 nm [61]. The activity of ascorbate peroxidase (APX) was determined by measuring the decrease in absorbance at 290 nm [61].

### ROS and NO fluorescence assays

The intracellular level of ROS in tomato roots was determined by treating the root tips with 20 μM 2′,7′-dichlorofluorescin diacetate (H2DCFDA) from Calbiochem (La Jolla, CA, USA) in a pH 7.8 HEPES buffer (20 mM) at 37°C for 30 minutes in the absence of light. Subsequently, the roots underwent a triple washing procedure in the same buffer for 15 minutes [62]. No fluorescence was detected in tomato root tips incubated in a pH 7.4, Tris–HCl buffer (10 mM) with 10 μM DAF-FM at 25°C for 1 hour in darkness. Subsequently, the fluorescence of ROS and NO was analysed by fluorescence microscopy. The quantitative analysis of fluorescence intensity was performed using ImageJ software from the National Institutes of Health in Bethesda, MD, USA.

### The S-nitrosation analysis of SlTrxh in tomato seedlings

After treatment with nitrate, tomato seedlings were harvested, quickly frozen, and then crushed using a mortar. The S-nitration levels were assessed using the Biotin switch technique (BST) method [63]. Protein extraction was performed using the HEN buffer. Following centrifugation at 14000 *g* for 15 minutes at 4°C, the protein concentration was adjusted and then treated with a freshly prepared mixture of MMTS and SDS for additional incubation at 50°C for 20 minutes. The MMTS was subsequently removed by acetone precipitation [64]. The pellets were resuspended in 10 μL of HENS buffer with 1% SDS, mixed with 1 mM biotin-HPDP and 1 mM ascorbate. The labeling reactions were conducted in the dark at room temperature for 1.5 hours. Protein precipitation was achieved by adding acetone at −20°C for 20 minutes. Subsequently, the pellets were resuspended in 25 mM Tris–HCl buffer (pH 6.8) containing 1% SDS. Next, all S-nitrosated proteins were enriched and purified using streptavidin-agarose beads overnight at 4°C. The proteins were quantified using the Bradford assay and analysed by Western blot with anti-SlTrxh antibody.

### Mass spectrometric analysis of S-nitrosylation residues

The purified His-SlTrxh recombinant protein was initially treated with GSNO and labeled with biotin-HPDP. It was then enzymatically digested with trypsin and analysed by LC–MS/MS using a Thermo Scientific Q Exactive HF-X Hybrid Quadrupole-Orbitrap MS System.

### The S-nitrosation analysis of SlTrxh protein *in vitro*

The SlTrxh, SlTrxh^C54S^, SlTrxh^C98S^, and SlTrxh^C101S^ recombinant protein expression and purification were conducted as previously described [27]. The S-nitrosation analysis of SlTrxh protein *in vitro* was conducted as previously described with minor modifications [65]*.* Each supernatant (100 μL) was treated with varying concentrations of GSNO (250, 500, 1000, 2000 μM) for 30 minutes at room temperature in the absence of light. Afterwards, the proteins were incubated with HEPES buffer (300 μL) containing 25 mM HEPES at pH 7.7, 1 mM EDTA, 3.3% SDS, and 27 mM MMTS for 20 min at room temperature. Vortexing was periodically carried out to block non-nitrosated free cysteine residues. To differentiate between specific and non-specific nitrosations, a group of samples was treated with 1 mM reduced glutathione (GSH) instead of S-nitrosoglutathione (GSNO). Any remaining methyl methanethiosulfonate (MMTS) was eliminated through precipitation with chilled acetone, and the proteins were resuspended in 60 μL of buffer containing hydroxylamine (HENS). Biotinylation was accomplished by adding 1 mM ascorbate and 2 mM biotin-HPDP, followed by incubation at room temperature for one hour. The biotinylated proteins were then subjected to acetone precipitation and resuspended in an equal volume of HENS buffer. Lastly, the detection of biotinylated proteins was performed using Western blotting with an anti-biotin antibody. The details of all primers used can be found in Table S3 (see online supplementary material).

### SNOs content assay

The concentration of S-nitrosothiols (SNOs) was determined using the Saville-Griess analysis method with slight modifications [66]. The plant tissue powder was lysed in 600 μL of extraction buffer (50 mM Tris–HCl, pH 8.0, 150 mM NaCl, and 1 mM PMSF) for 20 minutes on ice, followed by centrifugation at 10000 *g* for 15 minutes at 4°C. The resulting supernatant (50 μL) was incubated in darkness for 20 minutes with an equal volume of 1% sulfanilamide, with or without the addition of 0.2% (w/v) HgCl_2_. Subsequently, 100 μL of 0.02% NED was added and incubated for 5 minutes. The SNOs concentration was determined spectrophotometrically at 540 nm.

### Luciferase transactivation activity assay

The coding sequence of *SlMYB86* was fused with the effector vector pCAMBIA1300, under the regulation of the CaMV35S promoter. The DNA sequences derived from the *SlTrxh* promoter were then inserted into the pRI101-LUC vector, acting as the reporter vector. Subsequently, the effector and reporter vectors were co-transformed into the leaves of 4-week-old *N. benthamiana* plants. Transcription activation was evaluated by fluorescence measurement using the Tanon 5200 Multi Chemiluminescent Imaging System (Tanon, China) with D-luciferin Firefly (Gold Biotechnology, St Louis, MO, USA). These experiments were conducted with a minimum of three biological replicates. The complete list of primers used can be found in Table S4 (see online supplementary material).

### Chromatin immunoprecipitation (ChIP)-qPCR analysis

ChIP-qPCR was conducted on leaves (0.5 g) from 4-week-old seedlings of 35S:SlMYB86-GFP tomato seedlings using the Simple ChIP Plus Sonication ChIP Kit (Cell Signaling Technology). The leaves were finely ground in liquid nitrogen and subsequently cross-linked with 1% formaldehyde (Sigma-Aldrich) at 4°C for 10 minutes. Chromatin was fragmented using a Diagenode Bioruptor Plus instrument to yield DNA fragments of approximately 300 bp. Immunoprecipitation was carried out using an Anti-GFP (Sigma) antibody. The DNA obtained from ChIP was utilized for qPCR analysis. The complete list of primer sequences for qPCR can be found in Table S5 (see online supplementary material).

### Electrophoretic mobility shift assay (EMSA)

The entire coding sequence of *SlMYB86*, lacking a stop codon, was fused with a His tag and inserted into the pET28a vector. Subsequently, the SlMYB86 protein was isolated by His-agarose affinity chromatography. The biotin labeled probes were the promoter of SlTrxh containing MYB binding sites C1 (5’-CAACTG-3′) and C2 (5’-TAACTG-3′). The non-biotinylated regions of the same sequences were used as competitors. EMSA was performed according to the guidelines provided by the Light Shift Chemiluminescent EMSA Kit (Thermo Fisher Scientific, Shanghai, China). The complete list of primer sequences can be found in Table S6 (see online supplementary material).

### Yeast one-hybrid assays (Y1H)

The coding sequence of *SlMYB86* was incorporated into the pGADT7 vector utilizing the *Nde*I and *Bam*HI restriction enzyme sites. Correspondingly, the DNA sequences of the *SlTrxh* promoter were integrated into the pAbAi vector at the *Hin*dIII and *Kpn*I restriction enzyme sites. The Y1H was conducted in accordance with the manufacturer’s instructions (Coolaber, China). The complete list of primers utilized can be found in Table S7 (see online supplementary material).

### Yeast two-hybrid assays (Y2H)

The interaction between SlTrxh and SlGrx9 was explored by inserting their coding sequences into the pGADT7 vector (AD) and pGBKT7 vector (BD), respectively. pGADT7-SlTrxh was as the prey and pGBKT7-SlGrx9 as the bait. The Y2H was performed using the Y2H Gold-GAL4 interaction proving kit (Coolaber, China). The two constructs, pGADT7-SlTrxh and pGBKT7-SlGrx9, were co-transformed into the yeast strain Y2H Gold (Coolaber) and cultured on selective media SD/−Leu/−Trp and SD/−Leu/−Trp/−His/−Ade. Furthermore, Y2H Gold yeast strains were co-transformed with pGADT7-T + pGBKT7-53, pGADT7-SlTrxh + BD, AD + pGBKT7-SlGrx9, and AD + BD, serving as positive and negative controls. The list of all primers used is provided in Table S8 (see online supplementary material).

### Luciferase complementation assay (LCA)

The pCAMBIA1300-35S-cLUC and pCAMBIA1300-35S-nLUC vectors were utilized to insert the coding sequence of SlTrxh and SlGrx9, resulting in the cLUC-SlTrxh and SlGrx9-nLUC constructs for the LCA assay. *Agrobacterium* GV3101 strains carrying the mentioned constructs were infiltrated into the leaves of 4-week-old *N. benthamiana* tobacco plants and incubated for 48–72 hours*.* To investigate the interaction of S-nitrosation of SlTrxh with SlGrx9, the 54th cysteine was mutated to Tryptophan or Serine with DpnI enzyme (Takara, China). Nitrate and GSNO were added in the LCA experiment. Subsequently, the leaves were sprayed with a solution of 1 mM D-luciferin Firefly (Gold Biotechnology, St Louis, MO, USA) for 5 minutes, and the luciferase signals in the infiltrated region were detected using the Tanon 5200 Multi Chemiluminescent Imaging System (Tanon, China). The relative fluorescence intensity was carried out using ImageJ software. The list of primers used can be found in Table S9 (see online supplementary material).

### Co-immunoprecipitation assays (Co-IP)

To perform co-immunoprecipitation assays, SlTrxh and SlGrx9 were tagged with GFP and FLAG tags, respectively, using the pCAMBIA1300-GFP and pCAMBIA1300-FLAG vectors. The properly constructed vectors were co-injected into the leaves of 4-week-old *N. benthamiana* plants and incubated for 48–72 hours. Subsequently, a Co-IP assay was conducted using the Beyotime Anti-Flag Affinity Gel Kit (Beyotime, China). All primers used can be found in Table S10 (see online supplementary material).

### Subcellular localization analysis

The coding sequence of *SlMYB86* was inserted into the pRI101-GFP vector to create the pRI101-GFP-SlMYB86 plasmid, which included a 35S promoter, and then transferred into the *A. tumefaciens* EHA105 strain. Subsequently, the *Agrobacterium* carrying the pRI101-GFP-SlMYB86 and pRI101-GFP constructs were infiltrated into the epidermal cells of 4-week-old *N. benthamiana* leaves. Fluorescence images were captured using a laser confocal microscope model Olympus FV1000 (Olympus, Tokyo, Japan). The list of primers used can be found in Table S11 (see online supplementary material).

### Statistical analysis

The experimental design involved testing and analysing the results for three biological replicates where each replicate was evaluated using three technical repetitions. The differences between treatments were assessed using a one-way ANOVA and Duncan’s multiple range test, in GraphPad Prism version 6.00 for Windows (GraphPad Software, La Jolla, CA, USA) and SPSS software (IBM, USA). Distinct letters represent statistically significant variances at a significance level of *P* < 0.05.

## Supplementary Material

Web_Material_uhae184

## Data Availability

The whole of the data used in this study are available in the publication and its supplementary information files.

## References

[1] Tilman D, Cassman KG, Matson PA. et al. Agricultural sustainability and intensive production practices. Nature. 2002;418:671–7 12167873 10.1038/nature01014

[2] Ju XT, Kou CL, Christie P. et al. Changes in the soil environment from excessive application of fertilizers and manures to two contrasting intensive cropping systems on the North China plain. Environ Pollut. 2007;145:497–506 16777292 10.1016/j.envpol.2006.04.017

[3] Yu L, Ma S, Zhang X. et al. Ancient rapid functional differentiation and fixation of the duplicated members in rice Dof genes after whole genome duplication. Plant J: Cell Mol Biol. 2021;108:1365–81 10.1111/tpj.15516 34585814

[4] Qin SQ, Quan Z, Ma J. et al. Regulating nitrate excess in lettuce-planted greenhouse soil with available carbon addition through irrigation. Environ Sci Pollut R. 2019;26:19241–9 10.1007/s11356-019-05125-x 31065989

[5] Xu HN, He XZ, Wang K. et al. Identification of early nitrate stress response genes in spinach roots by suppression subtractive hybridization. Plant Mol Biol Report. 2012;30:633–42

[6] Du J, Guo S, Sun J. et al. Proteomic and physiological analyses reveal the role of exogenous spermidine on cucumber roots in response to Ca(NO3)2 stress. Plant Mol Biol. 2018;97:1–21 29633167 10.1007/s11103-018-0721-1

[7] Hossain Z, Pillai BV-S, Gruber MY. et al. Transcriptome profiling of Brassica napus stem sections in relation to differences in lignin content. BMC Genomics. 2018;19:255 29661131 10.1186/s12864-018-4645-6 PMC5903004

[8] Zhang Y, Chen H, Li S. et al. Comparative physiological and proteomic analyses reveal the mechanisms of Brassinolide-mediated tolerance to calcium nitrate stress in tomato. Front Plant Sci. 2021;12:724288 34868110 10.3389/fpls.2021.724288 PMC8636057

[9] Kolbert Z, Barroso JB, Brouquisse R. et al. A forty year journey: the generation and roles of NO in plants. Nitric Oxide: Biol Chem. 2019;93:53–70 10.1016/j.niox.2019.09.006 31541734

[10] Kwon E, Feechan A, Yun B-W. et al. AtGSNOR1 function is required for multiple developmental programs in Arabidopsis. Planta. 2012;236:887–900 22767201 10.1007/s00425-012-1697-8

[11] Yu MD, Lamattina L, Spoel SH. et al. Nitric oxide function in plant biology: a redox cue in deconvolution. New Phytol. 2014;202:1142–56 24611485 10.1111/nph.12739

[12] Fancy NN, Bahlmann AK, Loake GJ. Nitric oxide function in plant abiotic stress. Plant Cell Environ. 2017;40:462–72 26754426 10.1111/pce.12707

[13] Astier J, Kulik A, Koen E. et al. Protein S-nitrosylation: what's going on in plants? Free Radic Biol Med. 2012;53:1101–10 22750205 10.1016/j.freeradbiomed.2012.06.032

[14] Feng J, Chen LC, Zuo JR. Protein S-Nitrosylation in plants: current progresses and challenges. J Integr Plant Biol. 2019;61:1206–23 30663237 10.1111/jipb.12780

[15] Sircar E, Stoyanovsky DA, Billiar TR. et al. Analysis of glutathione mediated S-(de)nitrosylation in complex biological matrices by immuno-spin trapping and identification of two novel substrates. Nitric Oxide: Biol Chem. 2022;118:26–30 10.1016/j.niox.2021.10.008 PMC8688327 34742907

[16] Corpas FJ, Barroso JB. Peroxynitrite (ONOO) is endogenously produced in arabidopsis peroxisomes and is overproduced under cadmium stress. Ann Bot-London. 2014;113:87–96 10.1093/aob/mct260 PMC3864731 24232384

[17] Rasool G, Buchholz G, Yasmin T. et al. Overexpression of SlGSNOR impairs in vitro shoot proliferation and developmental architecture in tomato but confers enhanced disease resistance. J Plant Physiol. 2021;261:153433 33990008 10.1016/j.jplph.2021.153433

[18] Feechan A, Kwon E, Yun BW. et al. A central role for S-nitrosothiols in plant disease resistance. Proc Natl Acad Sci USA. 2005;102:8054–9 15911759 10.1073/pnas.0501456102 PMC1142375

[19] Chen YY, Huang YF, Khoo KH. et al. Mass spectrometry-based analyses for identifying and characterizing S-nitrosylation of protein tyrosine phosphatases. Methods. 2007;42:243–9 17532511 10.1016/j.ymeth.2007.03.002

[20] Lee SJ, Lee JR, Kim YH. et al. Investigation of tyrosine nitration and nitrosylation of angiotensin II and bovine serum albumin with electrospray ionization mass spectrometry. Rapid Commun Mass Spectrom: RCM. 2007;21:2797–804 17661312 10.1002/rcm.3145

[21] Shelar SB, Kaminska KK, Reddy SA. et al. Thioredoxin-dependent regulation of AIF-mediated DNA damage. Free Radic Biol Med. 2015;87:125–36 26119781 10.1016/j.freeradbiomed.2015.06.029

[22] Li H, Wan A. Apoptosis of rheumatoid arthritis fibroblast-like synoviocytes: possible roles of nitric oxide and the thioredoxin 1. Mediat Inflamm. 2013;2013:953462 10.1155/2013/953462 PMC3649754 23690674

[23] Haendeler J, Hoffmann J, Zeiher AM. Redox regulatory and anti-apoptotic functions of thioredoxin depend on S-nitrosylation at cysteine 69. Circulation. 2002;106:212–3 10.1038/ncb851 12244325

[24] Bashandy T, Guilleminot J, Vernoux T. et al. Interplay between the NADP-linked thioredoxin and glutathione systems in Arabidopsis auxin signaling. Plant Cell. 2010;22:376–91 20164444 10.1105/tpc.109.071225 PMC2845418

[25] Calderon A, Sánchez-Guerrero A, Ortiz-Espín A. et al. Lack of mitochondrial thioredoxin o1 is compensated by antioxidant components under salinity in *Arabidopsis thaliana* plants. Physiol Plant. 2018;164:251–67 29446456 10.1111/ppl.12708

[26] Sun LJ, Ren H, Liu R. et al. An h-type thioredoxin functions in tobacco defense responses to two species of viruses and an abiotic oxidative stress. Mol Plant Microbe In. 2010;23:1470–85 10.1094/MPMI-01-10-0029 20923353

[27] Wu FW, Nie L, Yu KN. et al. Characteristics of three thioredoxin genes and their role in chilling tolerance of harvested Banana fruit. Int J Mol Sci. 2016;17:1526 27618038 10.3390/ijms17091526 PMC5037801

[28] Ji MG, Park HJ, Cha J-Y. et al. Expression of *Arabidopsis thaliana* thioredoxin-h2 in *Brassica napus* enhances antioxidant defenses and improves salt tolerance. Plant Physiol Bioch. 2020;147:313–21 10.1016/j.plaphy.2019.12.032 31901883

[29] Salava H, Thula S, Mohan V. et al. Application of genome editing in tomato breeding: mechanisms, advances, and prospects. Int J Mol Sci. 2021;22:682 33445555 10.3390/ijms22020682 PMC7827871

[30] Chandrasekaran M, Boopathi T, Paramasivan M. A status-quo review on CRISPR-Cas9 gene editing applications in tomato. Int J Biol Macromol. 2021;190:120–9 34474054 10.1016/j.ijbiomac.2021.08.169

[31] García-Caparrós P . Breeding for yield quality parameters and abiotic stress in tomato using genome editing. In: Ricroch A. et al., eds. A Roadmap for Plant Genome Editing. Switzerland: Springer Nature, 2024,395–409

[32] Zhai J, Qi Q, Wang M. et al. Overexpression of tomato thioredoxin h (SlTrxh) enhances excess nitrate stress tolerance in transgenic tobacco interacting with SlPrx protein. Plant Sci. 2022;315:111137 35067307 10.1016/j.plantsci.2021.111137

[33] Ji R, Min J, Wang Y. et al. The role of plant growth regulators in modulating root architecture and tolerance to high-nitrate stress in tomato. Front Plant Sci. 2022;13:864285 35463444 10.3389/fpls.2022.864285 PMC9023760

[34] Foyer CH, Noctor G. Redox homeostasis and antioxidant signaling: a metabolic interface between stress perception and physiological responses. Plant Cell. 2005;17:1866–75 15987996 10.1105/tpc.105.033589 PMC1167537

[35] Meyer AJ, Dreyer A, Ugalde JM. et al. Shifting paradigms and novel players in Cys-based redox regulation and ROS signaling in plants – and where to go next. Biol Chem. 2021;402:399–423 33544501 10.1515/hsz-2020-0291

[36] Kaur H, Kaur H, Kaur H. et al. The beneficial roles of trace and ultratrace elements in plants. Plant Growth Regul. 2023;100:219–36

[37] Ali F, Li YH, Li FG. et al. Genome-wide characterization and expression analysis of cystathionine beta-synthase genes in plant development and abiotic stresses of cotton (Gossypium spp.). Int J Biol Macromol. 2021;193:823–37 34687765 10.1016/j.ijbiomac.2021.10.079

[38] Luan J, Dong J, Song X. et al. Overexpression of *Tamarix hispida* ThTrx5 confers salt tolerance to Arabidopsis by activating stress response signals. Int J Mol Sci. 2020;21:1165 32050573 10.3390/ijms21031165 PMC7037472

[39] Duan XH, Wang Z, Zhang Y. et al. Overexpression of a thioredoxin-protein-encoding gene, MsTRX, from *Medicago sativa* enhances salt tolerance to transgenic tobacco. Agronomy-Basel. 2022;12:1467

[40] Gietler M, Nykiel M, Orzechowski S. et al. Proteomic analysis of S-nitrosylated and S-glutathionylated proteins in wheat seedlings with different dehydration tolerances. Plant Physiol Biochem : PPB. 2016;108:507–18 27596017 10.1016/j.plaphy.2016.08.017

[41] Daurelio LD, Romero MS, Petrocelli S. et al. Characterization of *Citrus sinensis* transcription factors closely associated with the non-host response to *Xanthomonas campestris* pv. Vesicatoria. J Plant Physiol. 2013;170:934–42 23453188 10.1016/j.jplph.2013.01.011

[42] Hurali DT, Bhurta R, Tyagi S. et al. Analysis of NIA and GSNOR family genes and nitric oxide homeostasis in response to wheat-leaf rust interaction. Sci Rep. 2022;12:803 35039546 10.1038/s41598-021-04696-5 PMC8764060

[43] Kumar A, Dubey AK, Kumar V. et al. Over-expression of chickpea glutaredoxin (CaGrx) provides tolerance to heavy metals by reducing metal accumulation and improved physiological and antioxidant defence system. Ecotoxicol Environ Saf. 2020;192:110252 32014725 10.1016/j.ecoenv.2020.110252

[44] Kang BC, Wu Q, Sprague S. et al. Ectopic overexpression of an Arabidopsis monothiol glutaredoxin AtGRXS17 affects floral development and improves response to heat stress in chrysanthemum (chrysanthemum morifolium Ramat.). Environ Exp Bot. 2019;167:103864

[45] Ning X, Sun Y, Wang C. et al. A Rice CPYC-type Glutaredoxin *OsGRX20* in protection against bacterial blight, methyl viologen and salt stresses. Front Plant Sci. 2018;9:111 29479359 10.3389/fpls.2018.00111 PMC5811478

[46] Ströher E, Grassl J, Carrie C. et al. Glutaredoxin S15 is involved in Fe-S cluster transfer in mitochondria influencing lipoic acid-dependent enzymes, plant growth, and arsenic tolerance in Arabidopsis. Plant Physiol. 2016;170:1284–99 26672074 10.1104/pp.15.01308 PMC4775112

[47] Wang M, Hao J, Chen X. et al. SlMYB102 expression enhances low-temperature stress resistance in tomato plants. PeerJ. 2020;8:e10059 33083130 10.7717/peerj.10059 PMC7547593

[48] Jahan MA, Harris B, Lowery M. et al. Glyceollin transcription factor GmMYB29A2 regulates soybean resistance to phytophthora sojae. Plant Physiol. 2020;183:530–46 32209590 10.1104/pp.19.01293 PMC7271783

[49] Zhang X, Chen L, Shi Q. et al. SlMYB102, an R2R3-type MYB gene, confers salt tolerance in transgenic tomato. Plant Sci. 2020;291:110356 31928668 10.1016/j.plantsci.2019.110356

[50] Liu ZY, Li XP, Zhang TQ. et al. Overexpression of ThMYB8 mediates salt stress tolerance by directly activating stress-responsive gene expression. Plant Sci. 2021;302:110668 33288032 10.1016/j.plantsci.2020.110668

[51] Zhao K, Cheng Z, Guo Q. et al. Characterization of the poplar R2R3-MYB gene family and over-expression of PsnMYB108 confers salt tolerance in transgenic tobacco. Front Plant Sci. 2020;11:571881 33178243 10.3389/fpls.2020.571881 PMC7596293

[52] Zhang P, Wang R, Yang X. et al. The R2R3-MYB transcription factor AtMYB49 modulates salt tolerance in Arabidopsis by modulating the cuticle formation and antioxidant defence. Plant Cell Environ. 2020;43:1925–43 32406163 10.1111/pce.13784

[53] Song Y, Yang W, Fan H. et al. TaMYB86B encodes a R2R3-type MYB transcription factor and enhances salt tolerance in wheat. Plant Sci. 2020;300:110624 33180704 10.1016/j.plantsci.2020.110624

[54] Kim W-N, Kim H-J, Chung Y-S. et al. Construction of multiple guide RNAs in CRISPR/Cas9 vector using stepwise or simultaneous Golden Gate cloning: case study for targeting the FAD2 and FATB multigene in soybean. Plan Theory. 2021;10:2542 10.3390/plants10112542 PMC8622832 34834905

[55] Zheng P, Bai XG, Long J. et al. Nitric oxide enhances the nitrate stress tolerance of spinach by scavenging ROS and RNS. Sci Hortic-Amsterdam. 2016;213:24–33

[56] Kong FY, Deng Y, Zhou B. et al. A chloroplast-targeted DnaJ protein contributes to maintenance of photosystem II under chilling stress. J Exp Bot. 2014;65:143–58 24227338 10.1093/jxb/ert357 PMC3883286

[57] Qi Q, Yanyan D, Yuanlin L. et al. Overexpression of SlMDHAR in transgenic tobacco increased salt stress tolerance involving S-nitrosylation regulation. Plant Sci: Int J Exp Plant Biol. 2020;299:110609 10.1016/j.plantsci.2020.110609 32900447

[58] Rao KVM, Sresty TVS. Antioxidative parameters in the seedlings of pigeonpea (*Cajanus cajan* (L.) Millspaugh) in response to Zn and Ni stresses. Plant Sci. 2000;157:113–28 10940475 10.1016/s0168-9452(00)00273-9

[59] Gay CA, Gebicki JM. Measurement of protein and lipid hydroperoxides in biological systems by the ferric-xylenol orange method. Anal Biochem. 2003;315:29–35 12672409 10.1016/s0003-2697(02)00606-1

[60] Madhava Rao KV, Sresty TV. Antioxidative parameters in the seedlings of pigeonpea (*Cajanus cajan* (L.) Millspaugh) in response to Zn and Ni stresses. Plant Sci. 2000;157:113–28 10940475 10.1016/s0168-9452(00)00273-9

[61] Cakmak I, Marschner H. Magnesium deficiency and high light intensity enhance activities of superoxide dismutase, ascorbate peroxidase, and glutathione reductase in bean leaves. Plant Physiol. 1992;98:1222–7 16668779 10.1104/pp.98.4.1222 PMC1080336

[62] Mazel A, Leshem Y, Tiwari BS. et al. Induction of salt and osmotic stress tolerance by overexpression of an intracellular vesicle trafficking protein AtRab7 (AtRabG3e). Plant Physiol. 2004;134:118–28 14657401 10.1104/pp.103.025379 PMC316292

[63] Astier J, Besson-Bard A, Lamotte O. et al. Nitric oxide inhibits the ATPase activity of the chaperone-like AAA+ ATPase CDC48, a target for S-nitrosylation in cryptogein signalling in tobacco cells. Biochem J. 2012;447:249–60 22835150 10.1042/BJ20120257

[64] Bradford MM . A rapid and sensitive method for the quantitation of microgram quantities of protein utilizing the principle of protein-dye binding. Anal Biochem. 1976;72:248–54 942051 10.1016/0003-2697(76)90527-3

[65] Serpa V, Vernal J, Lamattina L. et al. Inhibition of AtMYB2 DNA-binding by nitric oxide involves cysteine S-nitrosylation. Biochem Biophys Res Commun. 2007;361:1048–53 17686455 10.1016/j.bbrc.2007.07.133

[66] Frungillo L, de Oliveira JF, Saviani EE. et al. Modulation of mitochondrial activity by S-nitrosoglutathione reductase in *Arabidopsis thaliana* transgenic cell lines. Biochim Biophys Acta. 2013;1827:239–47 23201478 10.1016/j.bbabio.2012.11.011

