# Supplementary material for: SlTrxh functions downstream of SlMYB86 and positively regulates nitrate stress tolerance via S-nitrosation in tomato seedling
Source: Hortic Res. 2024 Jul 10;11(9):uhae184. doi: 10.1093/hr/uhae184 (PMC11374535; doi:10.1093/hr/uhae184)
Supplement: Web_Material_uhae184 [file web_material_uhae184.zip › supplemental materials2024.06.08.docx]

Supplementary Table S1. The primers used for Plasmid construction and transgenic tomato transformation.

| Gene | Forward primer | Reverse primer |
| --- | --- | --- |
| pCAMBIA1300-GFP-*SlTrxh* (*Sma*I) | tacgaacgatactcgaccccATGCAAGCTGCCAGTCTCGC | ctagagtcgacggatcccccTAAATCATTATCAATGATGTCCCGC |
| pCAMBIA1300-GFP-*SlMYB86* (*Sma*I)  pHEE401E-*SlMYB86* (*Bsa*I-HF) | tacgaacgatactcgaccccATGGGTCATCACTGCTGC  atatatggtctcgattgGTAAACAGAAAGTAAAAAGgttttagagctagaaatagc | ctagagtcgacggatcccccACAATCCCATGCAAGT  attattggtctcgaaacTGTTAGTGAAAATTCCACGcaatctcttagtcgactctac |

Supplementary Table S2. The primers used for qRT-PCR analysis of the SlTrxh RNAi tomato seedling.

| Gene | Forward primer | Reverse primer |
| --- | --- | --- |
| *SlActin* | CAGTTAAATCACGACCAGCAAGAT | TGTCCCTATTTACGAGGGTTATGC |
| *SlSOD* | CTTCTTCGCAGTTTGAACG | AACACAGCCTTGACACGA |
| *SlCAT* | CCTTACCTGTGCTGATTTCC | TGGGACTTAGGATTTGGCT |
| *SlAPX* | TTCTCCAGCTGGTACTTTGAT | GAAGTGCATAACTTCCCATCT |
| *SlTrxh* | ACGAGATATGCAGGTTCGAGG | CGGGATGAGCCCTTCAGTTC |
| *SlMYB86* | AAACTAGCTGGACTACAAAGGTGT | TCTTGTTCACTAAAACAGCCCC |
| *SlNTRB* | GCACGTACCAGCTACCAAGT | GCACATCACCAGCAGCAAAA |
| *SlTPX* | TTGGGCTGGAGCTTGATCTG | TTTCATCAGCACCGGAGACC |
| *SlNR* | CCGGATCATGGTTTTCCGGT | TTGTACCACCAAGCTTCCGC |
| *SlPrx* | AAGTCCGGTGGTCTAGGTGA | CCTGAACGTATTGCAACGCC |

Supplementary Table S3. The primers used for S-nitrosation analysis of SlTrxh protein *in vitro*.

| Gene | Forward primer | Reverse primer |
| --- | --- | --- |
| pET28a-*SlTrxh* | cgcggatccgaattcATGCAAGCTGCCAGTCTCGC | tgcggccgcaagcttTAAATCATTATCAATGATGTCCCGC |
| pET28a-*SlTrx*^C54S^  pET28a-*SlTrx*^C98S^  pET28a-*SlTrx*^C101S^ | CCATTTCCAAACCCCCAGCTGTTGGAAAAT  CATGGTCTGGTCCTTGTATTTTGATGGCGC  GTCCTTCTATTTTGATGGCGCAAGAACTTG | GGTTTGGAAATGGTATTTCTGGAGAGTACTT  GGACCAGACCATGTGGCATAGAAATCAATA  AAAATAGAAGGACCACACCATGTGGCATAG |

Supplementary Table S4. The primers used for Luciferase (LUC) assay.

| Gene | Forward primer | Reverse primer |
| --- | --- | --- |
| pCAMBIA1300-GFP-*SlMYB86* (*Sma*I)  pRI101-*SlTrxh pro*-LUC (*Hind*III, *Sal*I ) | tacgaacgatactcgaccccATGGGTCATCACTGCTGC  gccagtgccaagcttGGAAATACAACATGAAGGG | ctagagtcgacggatcccccACAATCCCATGCAAGT  gtcttccatgtcgacGACATCGAGAGAATGAGAG |

Supplementary Table S5. Primers for ChIP-qPCR analysis.

| Gene | Forward primer | Reverse primer |
| --- | --- | --- |
| C0 (negative control) | CGGAATTAGCGAAGAGG | CTATGAAGAAGAGGTACCGAT |
| C1 | GAAGGGCATTTTGGGTA | GATGCTTCCAATGCATCA |
| C2 | CTAGCTCATGATGAACTTCTTC | GAGAGCAGAAGCTGAGGC |

Supplemental Table S6. Primers for EMSA.

| Gene | Forward primer | Reverse primer |
| --- | --- | --- |
| Emsa-*SlTrxh pro*-C1-Biotin  Emsa-*SlTrxh pro*-C1  Emsa-*SlTrxh pro*-C1-mut-Biotin  Emsa-*SlTrxh pro*-C2-Biotin  Emsa-*SlTrxh pro*-C2  Emsa-*SlTrxh pro*-C2-mut-Biotin | TTCTCAATGAAGAGTCAACTGATCCTTTGGTAATAC  TTCTCAATGAAGAGTCAACTGATCCTTTGGTAATAC  TTCTCAATGAAGAGTCCCCCCATCCTTTGGTAATAC  CAAATGATTGAGATTTAACTGGATTGTGATGCATTG  CAAATGATTGAGATTTAACTGGATTGTGATGCATTG  CAAATGATTGAGATTCCCCCCGATTGTGATGCATTG | GTATTACCAAAGGATCAGTTGACTCTTCATTGAGAA  GTATTACCAAAGGATCAGTTGACTCTTCATTGAGAA  GTATTACCAAAGGATGGGGGGACTCTTCATTGAGAA  CAATGCATCACAATCCAGTTAAATCTCAATCATTTG  CAATGCATCACAATCCAGTTAAATCTCAATCATTTG  CAATGCATCACAATCGGGGGGAATCTCAATCATTTG |

Supplemental Table S7. Primers for Y1H.

| Gene | Forward primer | Reverse primer |
| --- | --- | --- |
| pGADT7-*SlMYB86* (*Nde*I, *BamH*I) | gattacgctcatatgATGGGTCATCACTGCTGC | gagctcgatggatccACAATCCCATGCAAGT |
| *SlTrxh pro*-C2-pAbAi (*Hind*III, *Kpn*I) | tgaattgaaaagcttGGTCAGAGCAAGCACGAA | agatccccgggtaccGGTAGTGGCTGGGTCAAG |

Supplemental Table S8. Primers for Y2H.

| Gene | Forward primer | Reverse primer |
| --- | --- | --- |
| pGADT7-*SlTrxh* (*Nde*I, *BamH*I) | gattacgctcatatgATGCAAGCTGCCAGTCT | gagctcgatggatccTCATAAATCATTATCAATGATGT |
| pGBKT7-*SlGrx9* (*Nde*I, *BamH*I) | gaggacctgcatatgATGTCGCTCGGAATTTC | caggtcgacggatccTCAGGAGCACAATGTCCT |

Supplemental Table S9. Primers for LCA.

| Gene | Forward primer | Reverse primer |
| --- | --- | --- |
| pCAMBIA1300-35S-cLUC-*SlTrxh* (*Kpn*I, *Sal*I) | tcccggggcggtaccATGCAAGCTGCCAGTCT | gctctgcaggtcgacTCATAAATCATTATCAATGATGT |
| pCAMBIA1300-35S-*SlGrx9*-nLUC (*Kpn*I, *Sal*I)  cLUC-*SlTrxh^C54S^*  cLUC-*SlTrxh^C54W^* | gacgagctcggtaccATGTCGCTCGGAATTTC  CCATTTCCAAACCCCCAGCTGTTGGAAAAT  CCATTTGGAAACCCCCAGCTGTTGGAAAAT | cgagatctggtcgacGGAGCACAATGTCCTTTC  GGTTTGGAAATGGTATTTCTGGAGAGTACTT  GGTTTCCAAATGGTATTTCTGGAGAGTACTT |

Supplemental Table S10. Primers for Co-IP.

| Gene | Forward primer | Reverse primer |
| --- | --- | --- |
| pCAMBIA1300-GFP-*SlTrxh* (*Sma*I) | tacgaacgatactcgaccccATGCAAGCTGCCAGTCTCGC | ctagagtcgacggatcccccTAAATCATTATCAATGATGTCCCGC |
| pCAMBIA1300-FLAG-*SlGrx9* (*Nde*I, *EcoR*I) | ctgttgatacatatgATGTCGCTCGGAATTTC | atcatcgatgaattcGGAGCACAATGTCCTTTC |

Supplemental Table S11. Primers for Subcellular localization analysis.

| Gene | Forward primer | Reverse primer |
| --- | --- | --- |
| pRI101-GFP-*SlMYB86* (*Sal*I, *EcoR*I) | gcagcggccgtcgacATGGGTCATCACTGCTGC | gttgattcagaattcACAATCCCATGCAAGT |

a


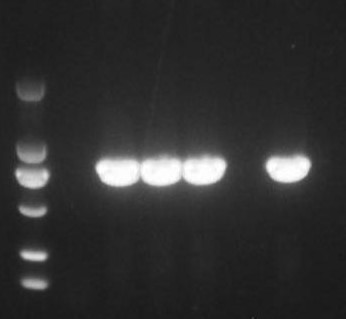


M

OE-1

WT

OE-2

OE-3

P

**－**

100

500

250

750

1000

2000

b


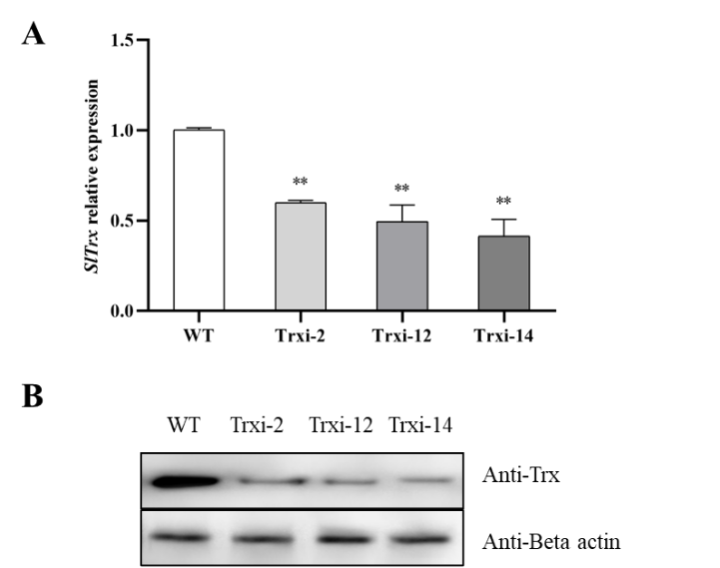


c

d

**Fig. S1 The characterization of *SlTrxh* overexpression and RNAi transgenic tomato. a** The genomic PCR analysis of *SlTrxh* overexpression transgenic tomato. **b** qRT-PCR analysis of *SlTrxh* overexpression transgenic tomato. c The qRT-PCR analysis of *SlTrxh* RNAi transgenic tomato. **d** Western blot analysis of *SlTrxh* RNAi transgenic tomato.


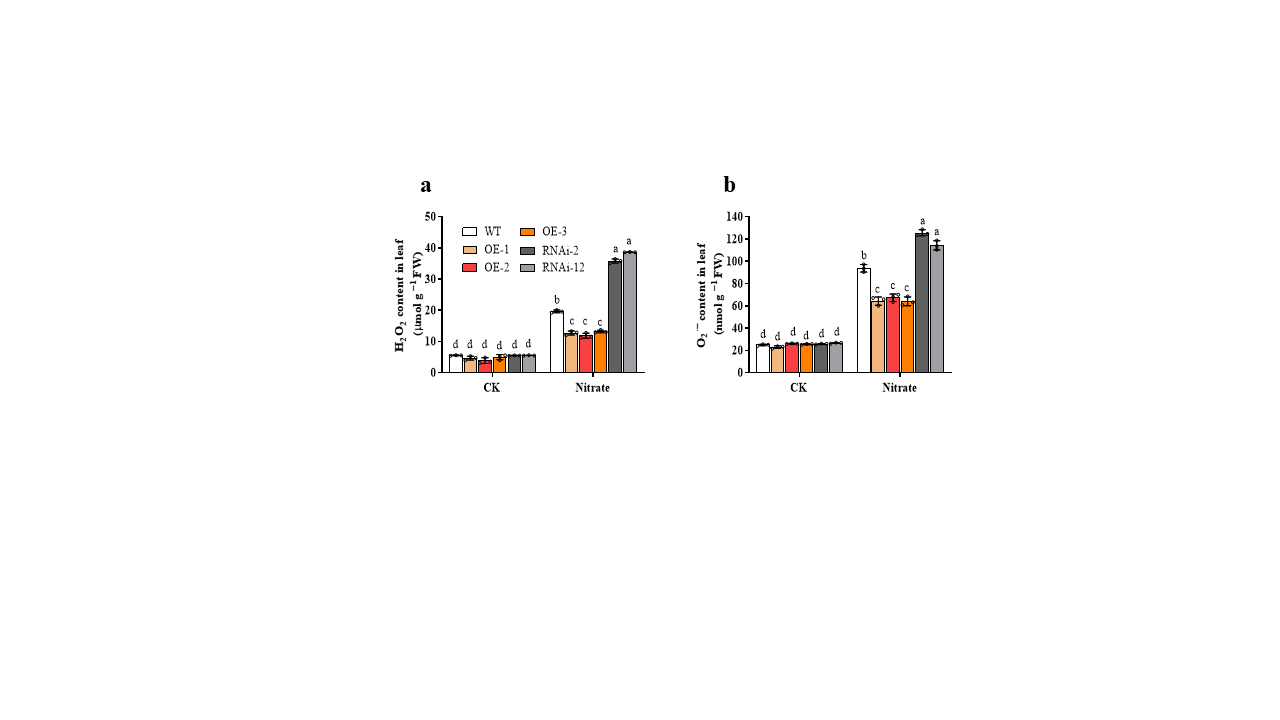


**Fig. S2 The H_2_O_2_ and O_2_·− contents in *SlTrxh* overexpressed (OE) and RNAi plants under nitrate stress.**

1. H_2_O_2_ content b. O_2_·− content. Data represent the mean ± SE (n = 3).


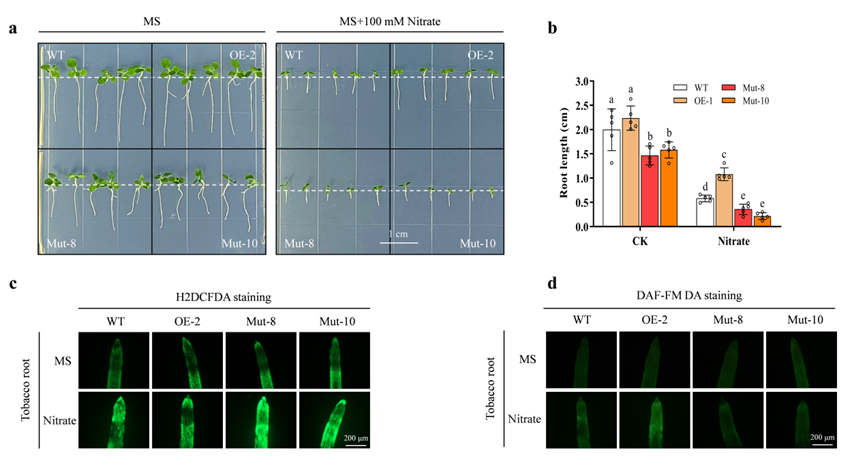


**Fig. S3 Cys54 serves as a crucial site for the SlTrxh's response to nitrate stress.**

**a** Phenotype of WT, *SlTrxh* overexpression and *SlTrxh^C54S^* mutation transgenic plants grown on excess nitrate stress. **b** Root length. **c** ROS dye H2DCFDA staining of primary root tips. Scale bar is 200 μm. **d** The effect of excess nitrate stress on NO accumulation—NO dye DAF-FM staining of primary root tips. Scale bar is 200 μm.


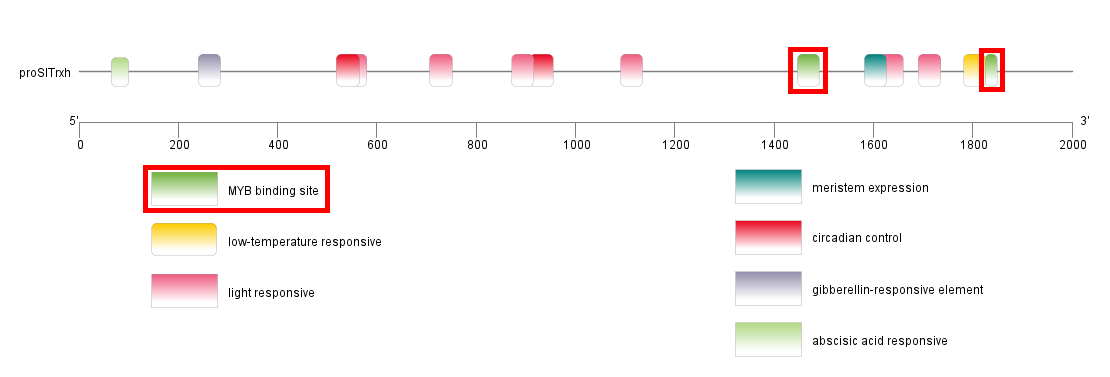


**Fig. S4 Analysis of cis-acting elements in SlTrxh promoter.**

The cis-acting element analysis of SlTrxh promoter. The MBS cis-regulatory elements are marked using red rectangles.a

a


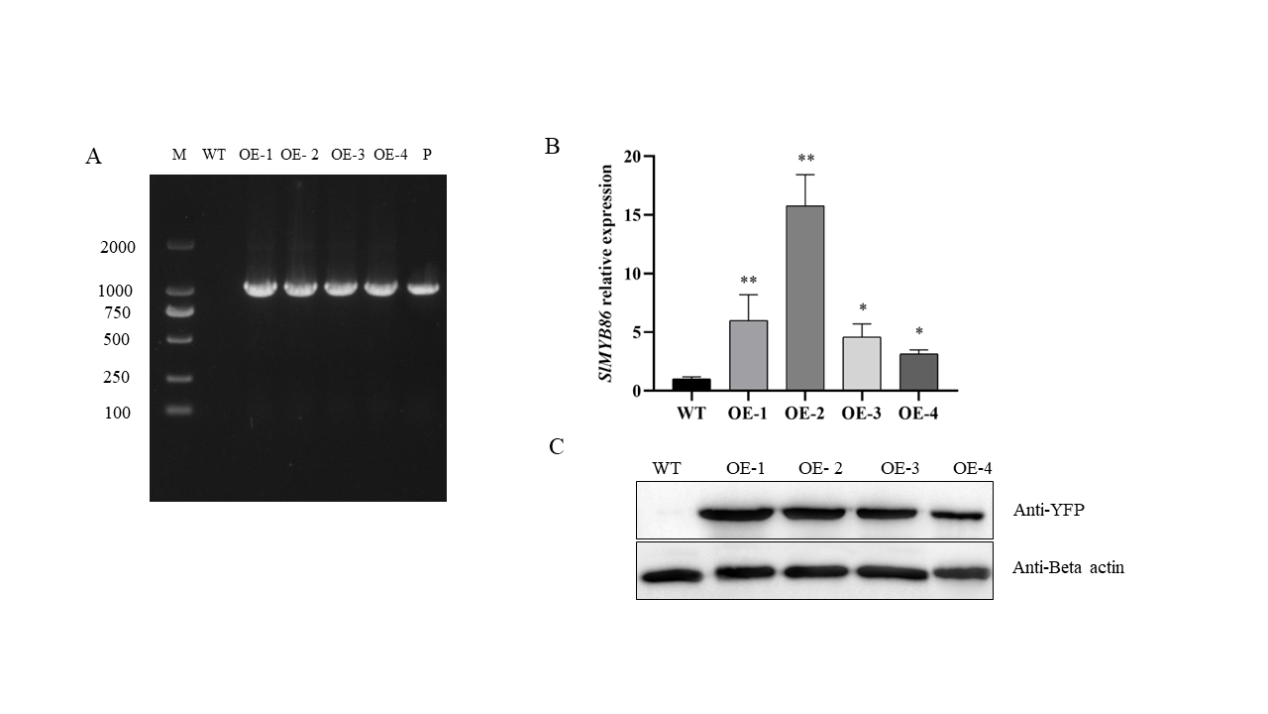


b


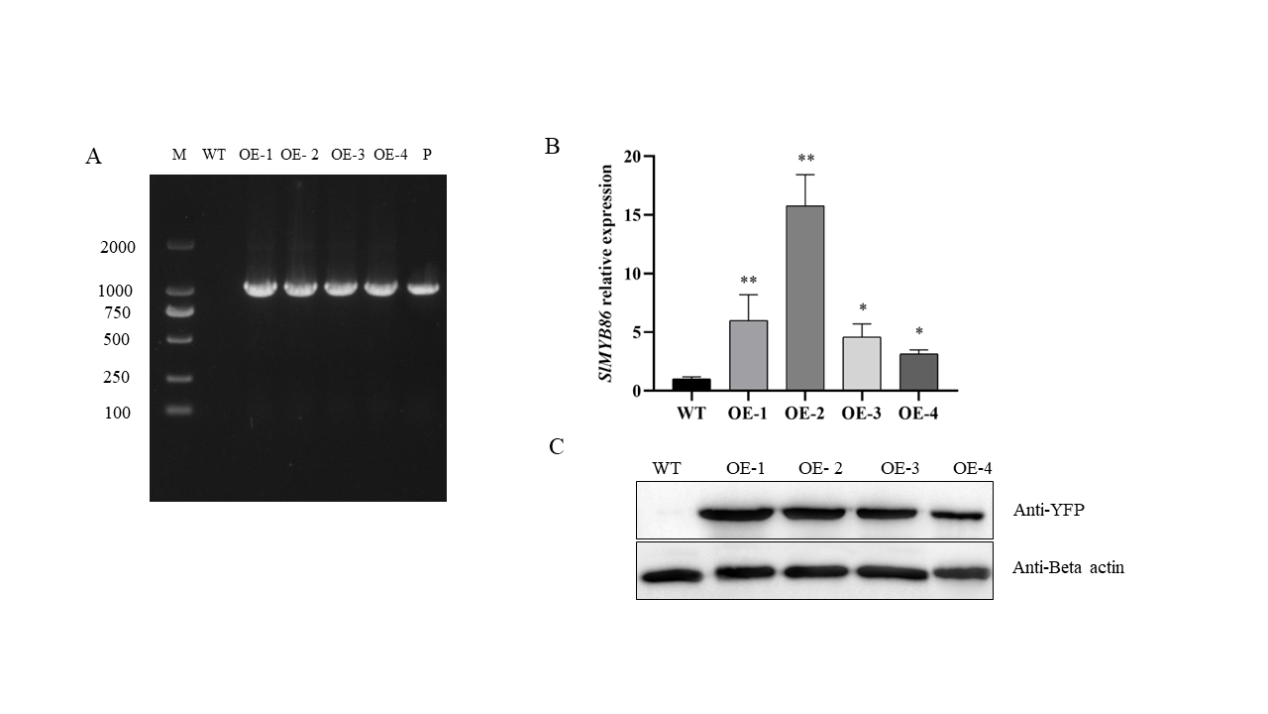


c

**Fig. S5The characterization of *SlMYB86* overexpression transgenic tomato.** **a** The genomic PCR analysis of *SlMYB86* overexpression transgenic tomato. **b** qRT-PCR analysis of *SlMYB86* overexpression transgenic tomato. **c** Western blot analysis of *SlMYB86* overexpression transgenic tomato.


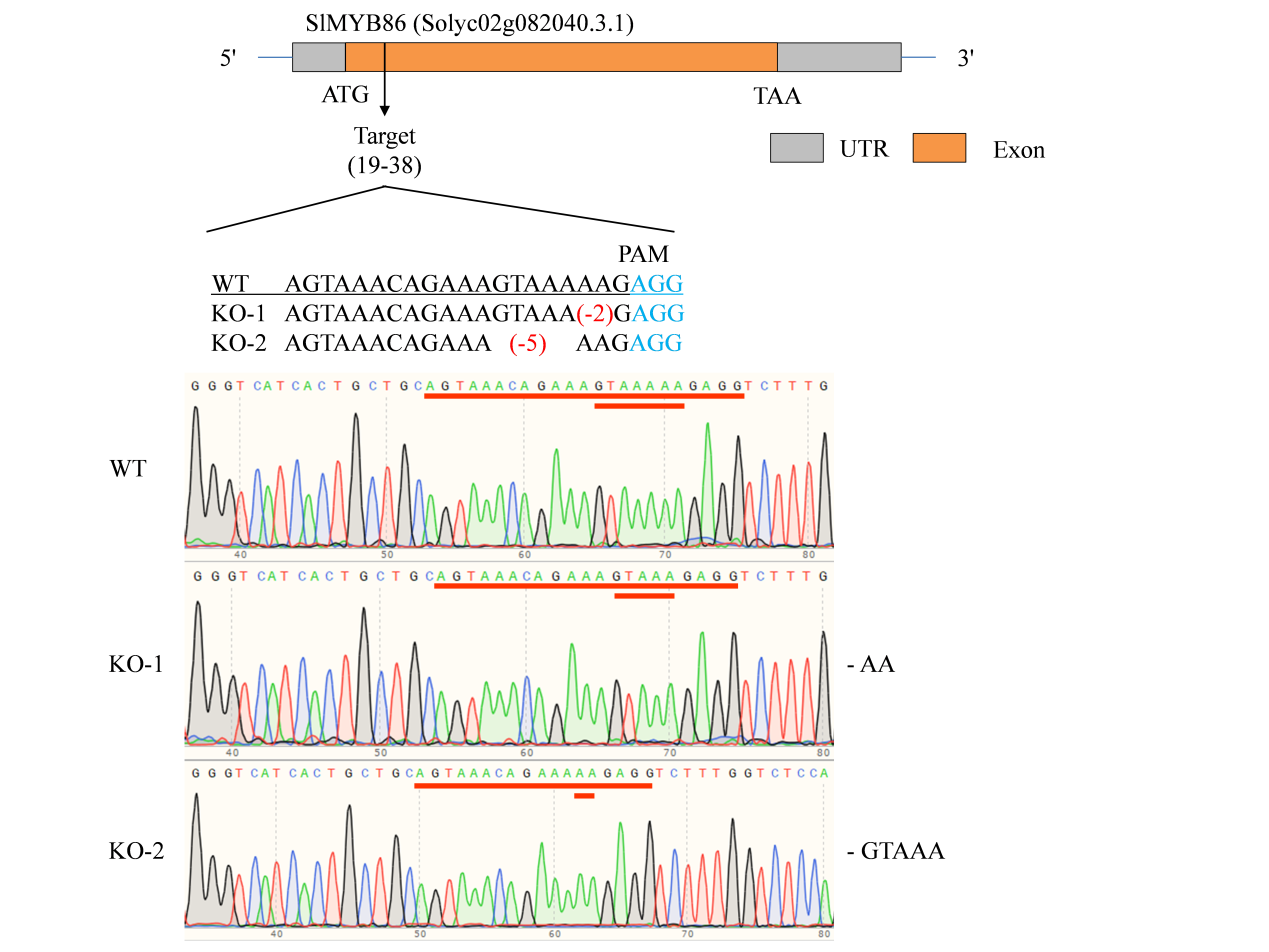


**Fig. S6** **The characterization of *slmyb86* knockout transgenic tomato by sequence analysis.**
